# Supplementary material for: Glycemic Challenge Is Associated with the Rapid Cellular Activation of the Locus Ceruleus and Nucleus of Solitary Tract: Circumscribed Spatial Analysis of Phosphorylated MAP Kinase Immunoreactivity
Source: J Clin Med. 2023 Mar 24;12(7):2483. doi: 10.3390/jcm12072483 (PMC10095283; doi:10.3390/jcm12072483)
Supplement: Supplementary file 1 [file jcm-12-02483-s001.zip › jcm-2181174-Supplemental Materials/Supplemental Figures S1¿CS5-27Mar23.pdf]

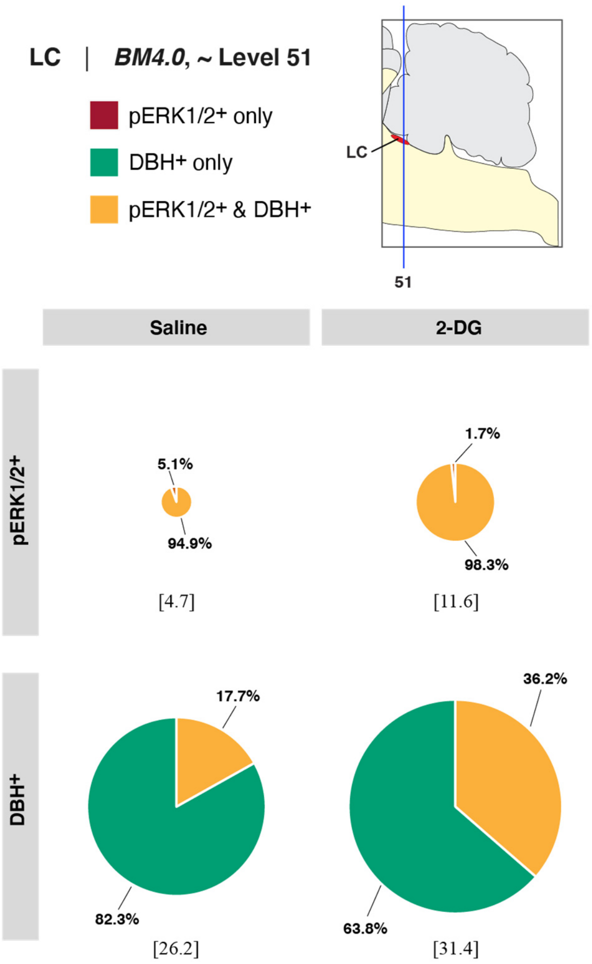

Figure S1. Relative percent differences of perikaryon phenotypes in *locus ceruleus (Wenzel & Wenzel, 1912)* [82] (LC) region in saline- and 2-DG-treated subjects. Relative percentages of single- and double-labeled perikaryon profiles were calculated for pERK1/2+ (*top row*) and DBH+ (*bottom row*) cell types in each treatment group and displayed as pie charts. Charts are paneled with treatment group as column and perikaryon type as row. The size of each pie chart was scaled according to its corresponding total Abercrombie-corrected [106] perikaryon profile count, which is noted below each pie chart as a numerical value in brackets.

Supplemental Figures for Tapia et al. (*J. Clin. Med.)*

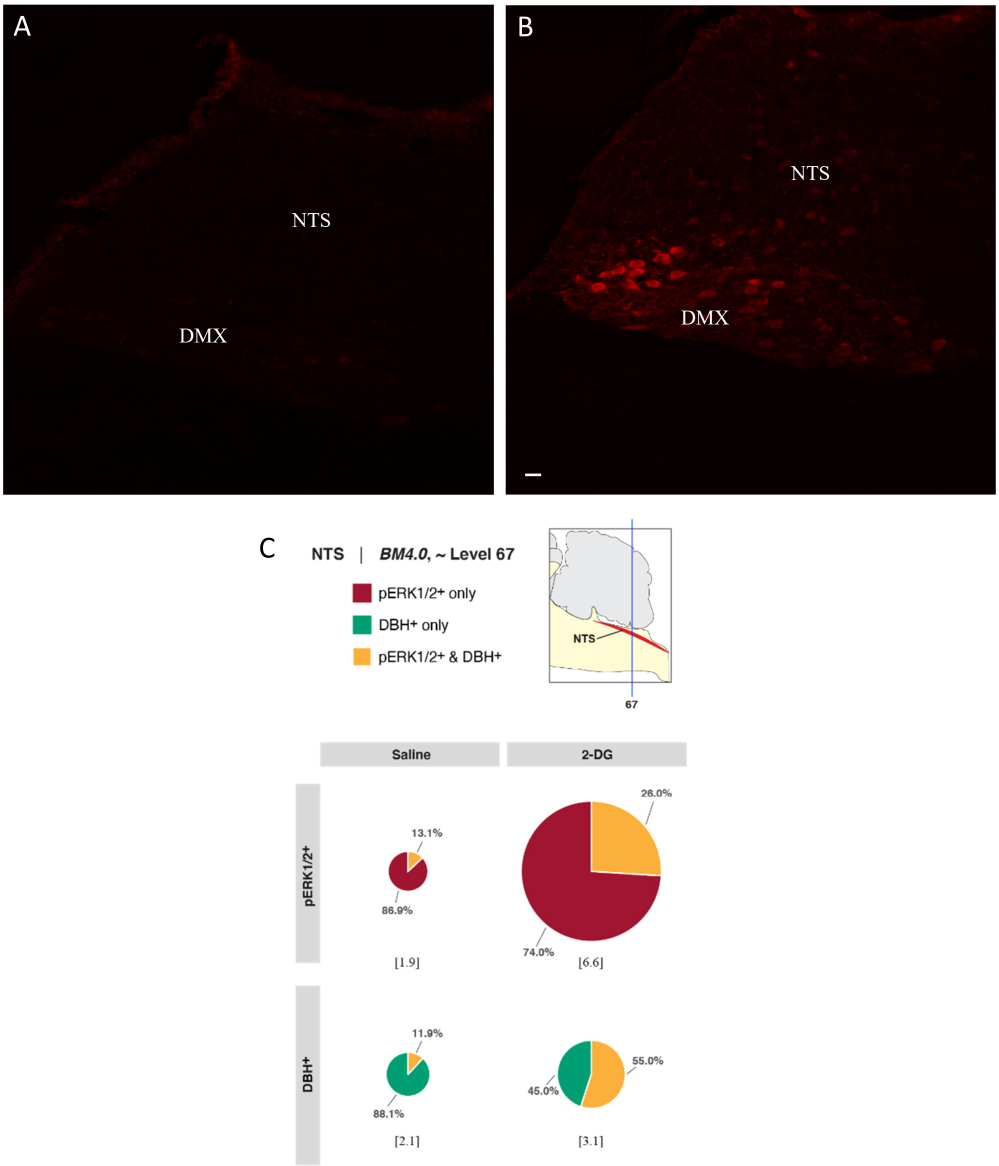

Figure S2. Activation of *nucleus of solitary tract (>1840)* (NTS) and relative percent differences of NTS perikaryon phenotypes in saline- and 2-DG-treated subjects. (**A**,**B**). Enlargements of the images in **Figure 5** of the main parent study (**5A**(**iii**) and **5B**(**iii**), respectively), showing an absence of elevations of phospho-ERK1/2 immunoreactive perikaryon profiles in the NTS of a saline-treated subject (**A**) as compared to a subject treated with intravenous 2-DG (**B**). The NTS is labeled in each panel to show differences in the presence of faintly-staining but clearly observable perikaryon profiles in the 2-DG-treated tissue (see **Figure 5** for exact boundaries of the NTS). The *dorsal motor nucleus of vagus (>1840)* (DMX) is also shown. Scale bar in **B** = 20 µm, and also applies to **A**. (**C**). Relative percent differences of perikaryon phenotypes in *BM4.0* atlas level 67-registered NTS in saline- and 2-DG-treated subjects. Relative percentages of single- and double-labeled perikaryon profiles were calculated for pERK1/2$^+$ (*top row*) and DBH$^+$ (*bottom row*) cell types in each treatment group and displayed as pie charts. Charts are paneled with treatment group as column and cell type as row. The size of each pie chart was scaled according to its corresponding total Abercrombie-corrected [106] perikaryon profile count (listed in brackets below each pie chart). For example, the 2-DG group pERK1/2$^+$ pie chart (*top right panel*) has a larger diameter than the saline group pERK1/2$^+$ pie chart (*top left panel*), corresponding to the Abercrombie-corrected perikaryon profile number presented in **Figure 6**. Note the high percentage of pERK1/2$^+$-only perikaryon profiles in the *top row* in both saline- and 2-DG-treated rats.

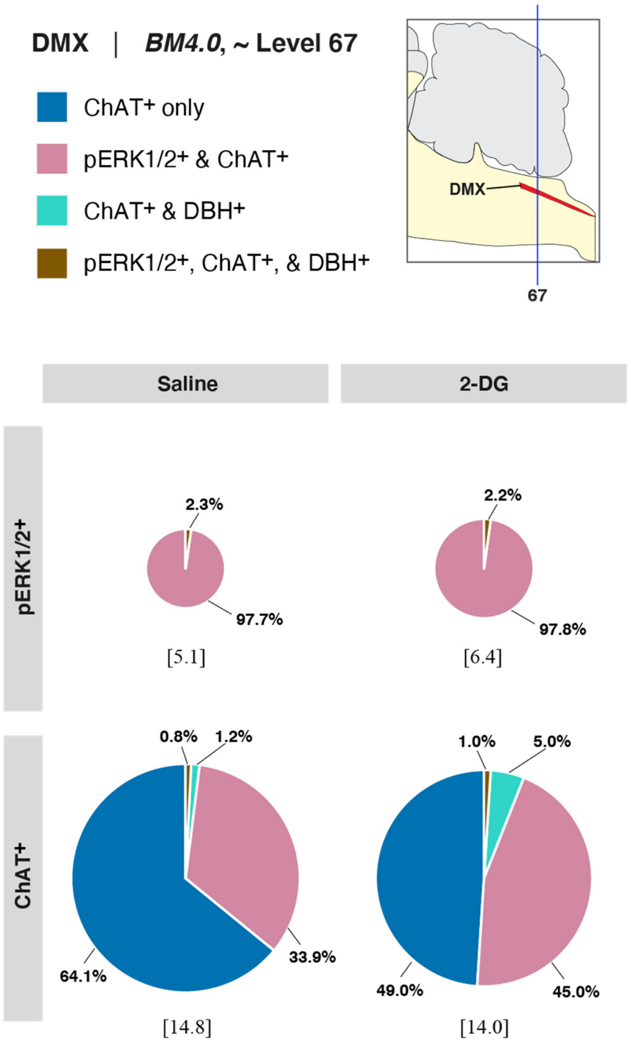

Figure S3. Relative percent differences of perikaryon phenotypes in *BM4.0* atlas level 67-registered *dorsal motor nucleus of vagus (>1840)* (DMX) in saline- and 2-DG-treated subjects. Relative percentages of single-, double-, and triple-labeled perikaryon profiles were calculated for pERK1/2$^+$ (*top row*) and ChAT$^+$ (*bottom row*) cell types in each treatment group and displayed as pie charts. Charts are paneled with treatment group as column and cell type as row. The size of each pie chart was scaled according to its corresponding total Abercrombie-corrected [106] perikaryon profile number.

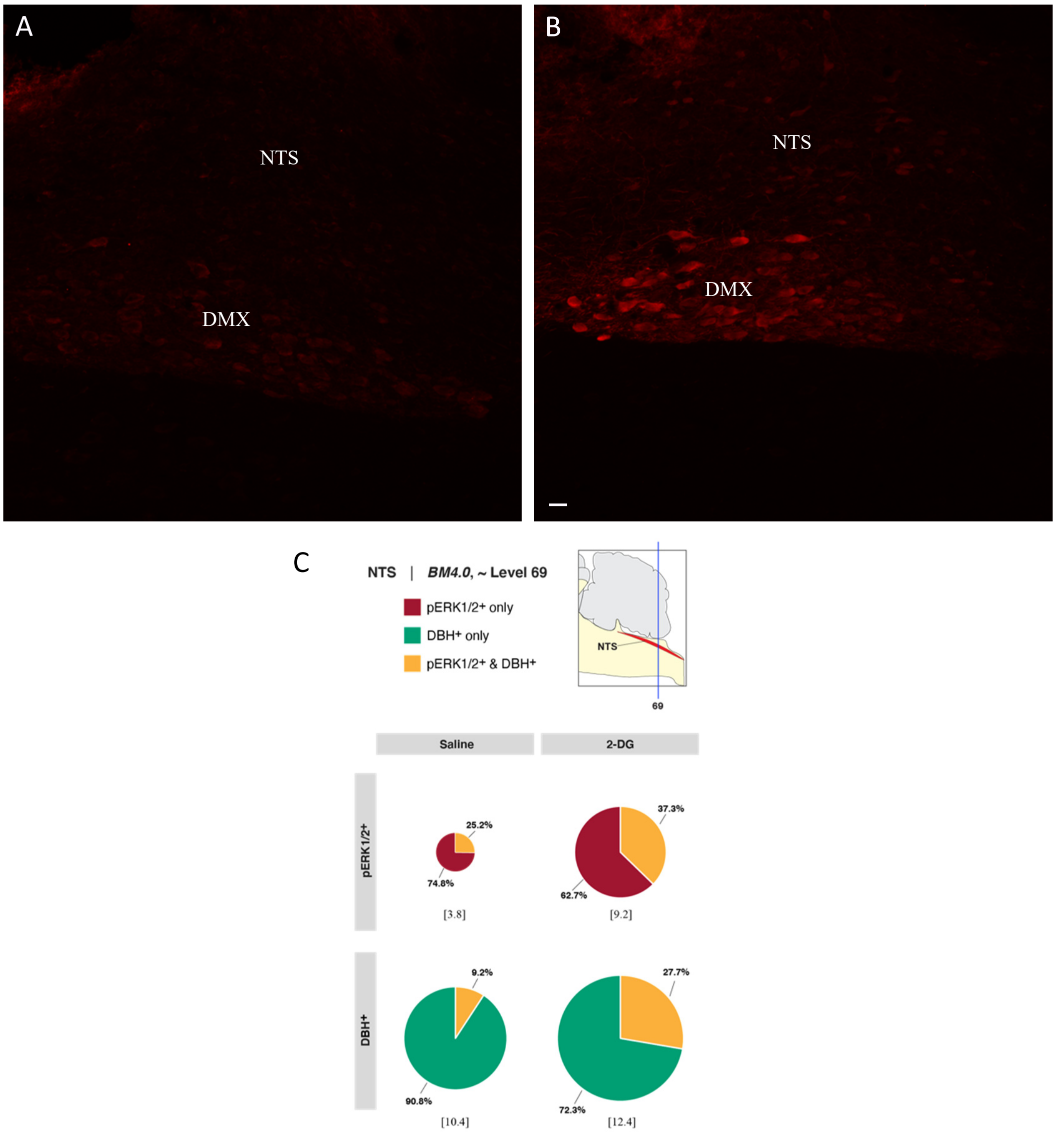

Figure S4. Activation of the *nucleus of solitary tract (>1840)* (NTS) (**A, B**) and relative percent changes of perikaryon profile phenotypes in *BM4.0* atlas level 69-registered NTS (**C**) 15 min after 2-DG administration. (**A,B**). Enlargements of the images in **Figure 8** of the main parent study (**8A**(**iii**) and **8B**(**iii**), respectively), showing an absence of elevations of phospho-ERK1/2 immunoreactive perikaryon profiles in the NTS of a saline-treated subject (**A**) as compared to a subject treated with intravenous 2-DG (**B**). The NTS is labeled in each panel to show differences in the presence of faintly-staining but clearly observable perikaryon profiles in the 2-DG-treated tissue (see **Figure 8** for exact boundaries of the NTS). The *dorsal motor nucleus of vagus (>1840)* (DMX) is also shown. Scale bar in **B** = 20 µm, and also applies to **A**. (**C**) Relative percentages of single and double-labeled perikaryon profiles were calculated for pERK1/2^{+} (*top row*) and DBH^{+} (*bottom row*) perikaryon types in each treatment group and displayed as pie charts. Charts are paneled with treatment group as column and perikaryon type as row. The size of each pie chart was scaled according to its corresponding total Abercrombie-corrected [106] perikaryon profile numbers.

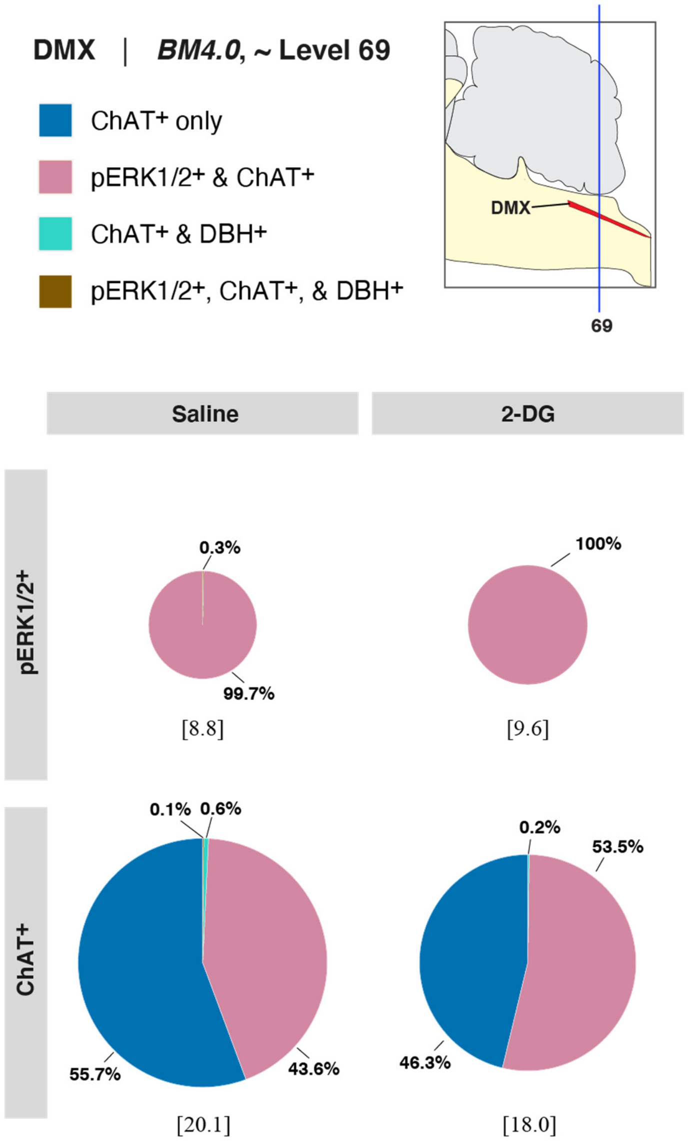

Figure S5. Relative percent changes of perikaryon phenotypes in the *BM4.0* atlas level 69-registered *dorsal motor nucleus of vagus nerve (>1840)* (DMX) 15 min after 2-DG administration. Relative percentages of single-, double-, and triple-labeled perikaryon profiles were calculated for pERK1/2$^+$ (*top row*) and ChAT$^+$ (*bottom row*) perikaryon types in each treatment group and displayed as pie charts. Charts are paneled with treatment group as column and perikaryon type as row. The size of each pie chart was scaled according to its corresponding total Abercrombie-corrected [106] perikaryon profile numbers.
