# Supplementary material for: Glycemic Challenge Is Associated with the Rapid Cellular Activation of the Locus Ceruleus and Nucleus of Solitary Tract: Circumscribed Spatial Analysis of Phosphorylated MAP Kinase Immunoreactivity
Source: J Clin Med. 2023 Mar 24;12(7):2483. doi: 10.3390/jcm12072483 (PMC10095283; doi:10.3390/jcm12072483)
Supplement: Supplementary file 1 [file jcm-12-02483-s001.zip › jcm-2181174-Supplemental Materials/Supplemental Tables S1¿CS6-10Mar23.pdf]

Supplemental Tables S1–S6 for Tapia et al. (*J. Clin. Med.)*

Table S1. Summary of descriptive statistics for LC perikaryon profiles at Atlas Level 51

Region	Perikaryon phenotype	Saline				2-DG			
		n	Median	Q1	Q3	n	Median	Q1	Q3
LC (~51)	pERK1/2$^+$	5	14.5	11.5	17	5	33	31.5	37.5
	DBH$^+$	5	73	73	86	5	97.5	92.5	97.5
	pERK1/2$^+$ & DBH$^-$	5	1	0.5	1	5	0.5	0.5	1
	pERK$^-$ & DBH$^+$	5	57	53	79	5	63.5	61.5	65
	pERK$^+$ & DBH$^+$	5	13	11	16	5	32.5	30.5	37.5

Table S2. Summary of non-parametric test statistics and effect sizes for LC perikaryon profiles at Atlas Level 51

Region	Perikaryon phenotype	Sample size		Wilcoxon Rank-Sum test			Wilcoxon effect size	
		Saline	2-DG	Test statistic	*p*-value	FDR-adjusted *p*-value	Effect size	Magnitude*
LC (~51)	pERK1/2$^+$	5	5	25	**0.01**	**0.03**	**0.83**	large
	DBH$^+$	5	5	21	0.09	0.22	0.56	large
	pERK1/2$^+$ & DBH$^-$	5	5	6.5	0.24	0.45	0.40	moderate
	pERK1/2$^-$ & DBH$^+$	5	5	14	0.83	0.45	0.10	small
	pERK1/2$^+$ & DBH$^+$	5	5	25	**0.01**	**0.03**	**0.83**	large

* 0.1 to < 0.3 – small, 0.3 to < 0.5 – moderate, and > or = 0.5 – large

Table S3. Summary of descriptive statistics for NTS and DMX perikaryon profiles at Atlas Level 67

Region	Perikaryon phenotype	Saline				2-DG			
		n	Median	Q1	Q3	n	Median	Q1	Q3
NTS (~67)	pERK1/2$^+$	6	1.7	1.2	2.5	5	6.1	6.1	7.2
	DBH$^+$	6	2.1	1.6	2.3	5	2.9	2.7	3.9
	pERK1/2$^+$ & DBH$^-$	6	1.6	0.9	2.0	5	4.2	4.0	5.5
	pERK1/2$^-$ & DBH$^+$	6	1.7	1.3	2.3	5	1.0	0.8	2.1
	pERK1/2$^+$ & DBH$^+$	6	0.2	0.2	0.3	5	1.9	1.7	2.1
DMX (~67)	pERK1/2$^+$	6	5.4	3.3	6.1	5	6.1	4.5	8.7
	ChAT$^+$	6	14.9	13.9	15.6	5	15.2	10.7	15.4
	DBH$^+$	6	0.3	0.2	0.3	5	0.9	0.7	1.0
	pERK1/2$^+$, ChAT$^-$, & DBH$^-$	6	0	0	0	5	0	0	0
	pERK1/2$^-$, ChAT$^+$, & DBH$^-$	6	9.4	8.1	10.7	5	6.5	5.2	8.5
	pERK1/2$^-$, ChAT$^-$, & DBH$^+$	6	0	0	0	5	0	0	0
	pERK1/2$^+$, ChAT$^+$	6	5.4	3.3	6.1	5	6.1	4.5	8.7
	pERK1/2$^+$, DBH$^+$	6	0	0	0	5	0	0	0
	ChAT$^+$, DBH$^+$	6	0.3	0.2	0.3	5	0.9	0.7	1.0
	pERK1/2$^+$, ChAT$^+$, & DBH$^-$	6	5.2	3.3	6.0	5	6.1	4.4	8.6
	pERK1/2$^-$, ChAT$^+$, & DBH$^+$	6	0.2	0.04	0.2	5	0.9	0.7	0.9
	pERK1/2$^+$, ChAT$^-$, & DBH$^+$	6	0	0	0	5	0	0	0
	pERK1/2$^+$, ChAT$^+$, & DBH$^+$	6	0.2	0.03	0.2	5	0.2	0	0.2

Table S4. Summary of non-parametric test statistics and effect sizes for NTS and DMX perikaryon profiles at Atlas Level 67

Region	Perikaryon phenotype	Sample size		Wilcoxon Rank-Sum test			Wilcoxon effect size	
		Saline	2-DG	Test statistic	*p*-value	FDR-adjusted *p*-value	Effect size	Magnitude *
NTS (~ 67)	pERK1/2$^+$	6	5	30	**<0.01**	**0.02**	0.83	large
	DBH$^+$	6	5	23	0.17	0.38	0.44	moderate
	pERK1/2$^+$ & DBH$^-$	6	5	30	**<0.01**	**0.02**	0.83	large
	pERK1/2$^-$ & DBH$^+$	6	5	10	0.41	0.67	0.28	small
	pERK1/2$^+$ & DBH$^+$	6	5	29	**0.01**	**0.02**	0.78	large
DMX (~67)	pERK1/2$^+$	6	5	18.5	0.58	0.71	0.19	small
	ChAT$^+$	6	5	14	0.93	1.00	0.05	small
	DBH$^+$	6	5	25.5	0.06	0.19	0.59	large
	pERK1/2$^+$, ChAT$^-$, & DBH$^-$	6	5	NA	NA	NA	NA	NA
	pERK1/2$^-$, ChAT$^+$, & DBH$^-$	6	5	4.5	0.07	0.19	0.58	large
	pERK1/2$^-$, ChAT$^-$, & DBH$^+$	6	5	NA	NA	NA	NA	NA
	pERK1/2$^+$, ChAT$^+$	6	5	18.5	0.58	0.71	0.19	small
	pERK1/2$^+$, DBH$^+$	6	5	NA	NA	NA	NA	NA
	ChAT$^+$, DBH$^+$	6	5	25.5	0.06	0.19	0.59	large
	pERK1/2$^+$, ChAT$^+$, & DBH$^-$	6	5	18.5	0.58	0.71	0.19	small
	pERK1/2$^-$, ChAT$^+$, & DBH$^+$	6	5	25	0.08	0.19	0.56	large
	pERK1/2$^+$, ChAT$^-$, & DBH$^+$	6	5	NA	NA	NA	NA	NA
	pERK1/2$^+$, ChAT$^+$, & DBH$^+$	6	5	16	0.92	1.00	0.06	small

* 0.1 to < 0.3 – small, 0.3 to < 0.5 – moderate, and > or = 0.5 – large

Table S5. Summary of descriptive statistics for NTS and DMX perikaryon profiles at atlas level 69

Region	Perikaryon phenotype	Saline				2-DG			
		n	Median	Q1	Q3	n	Median	Q1	Q3
NTS (~69)	pERK1/2^{+}	6	3.6	3.5	4.0	4	8.3	8.3	9.3
	DBH^{+}	6	9.7	9.3	10.4	4	12.6	11.3	13.7
	pERK1/2^{+} & DBH$^{–}$	6	2.9	2.2	3.0	4	5.7	5.3	6.1
	pERK1/2$^{–}$ & DBH^{+}	6	8.9	8.4	9.5	4	8.8	8.3	9.5
	pERK1/2^{+} & DBH^{+}	6	0.8	0.7	1.1	4	2.9	2.7	3.6
DMX (~69)	pERK1/2^{+}	6	8.8	8.0	9.6	4	8.5	6.9	11.2
	ChAT^{+}	6	20.9	20.3	21.4	4	17.0	16.2	18.8
	DBH^{+}	6	0.1	0	0.1	4	0	0	0.04
	pERK1/2^{+}, ChAT$^{–}$, & DBH$^{–}$	6	0	0	0	4	0	0	0
	pERK1/2$^{–}$, ChAT^{+}, & DBH$^{–}$	6	11.9	11.5	12	4	8.0	7.5	8.8
	pERK1/2$^{–}$, ChAT$^{–}$, & DBH^{+}	6	0	0	0	4	0	0	0
	pERK1/2^{+}, ChAT^{+}	6	8.8	8.0	9.6	4	8.5	6.9	11.2
	pERK1/2^{+}, DBH^{+}	6	0	0	0	4	0	0	0
	ChAT^{+}, DBH^{+}	6	0.8	0	0.1	4	0	0	0.04
	pERK1/2^{+}, ChAT^{+}, & DBH$^{–}$	6	8.0	9.6	1.6	4	6.9	11.2	4.4
	pERK1/2$^{–}$, ChAT^{+}, & DBH^{+}	6	0.8	0	0.1	4	0	0	0.04
	pERK1/2^{+}, ChAT$^{–}$, & DBH^{+}	6	0	0	0	4	0	0	0
	pERK1/2^{+}, ChAT^{+}, & DBH^{+}	6	0	0	0	4	0	0	0

Table S6. Summary of non-parametric test statistics and effect sizes for NTSm/DMX perikaryon profiles at Atlas Level 69

Region	Perikaryon phenotype	Sample size		Wilcoxon Rank-Sum test		Wilcoxon effect size	
		Saline	2-DG	Test statistic	*p*-value	Effect size	Magnitude*
NTSm (~ 69)	pERK1/2^{+}	6	4	24	**0.01**	0.82	large
	DBH^{+}	6	4	18	0.24	0.40	moderate
	pERK1/2^{+}, DBH$^{–}$	6	4	22.5	**0.03**	0.71	large
	pERK1/2$^{–}$, DBH+	6	4	11	0.91	0.07	small
	pERK1/2^{+}, DBH^{+}	6	4	24	**0.01**	0.82	large
DMX (~69)	pERK1/2^{+}	6	4	11.5	1.00	0.03	small
	ChAT^{+}	6	4	8	0.45	0.27	small
	DBH^{+}	6	4	8.5	0.46	0.27	small
	pERK1/2^{+}, ChAT$^{–}$, DBH$^{–}$	6	4	NA	NA	NA	NA
	pERK1/2$^{–}$, ChAT^{+}, DBH$^{–}$	6	4	3	0.07	0.60	large
	pERK1/2$^{–}$, ChAT$^{–}$, DBH^{+}	6	4	NA	NA	NA	NA
	pERK1/2^{+}, ChAT^{+}	6	4	11.5	1.00	0.03	small
	pERK1/2^{+}, DBH^{+}	6	4	NA	NA	NA	NA
	ChAT^{+}, DBH^{+}	6	4	8.5	0.46	0.27	small
	pERK1/2^{+}, ChAT^{+}, DBH$^{–}$	6	4	11.5	1.00	0.03	small
	pERK1/2$^{–}$, ChAT^{+}, DBH^{+}	6	4	8.5	0.46	0.27	small
	pERK1/2^{+}, ChAT$^{–}$, DBH^{+}	6	4	NA	NA	NA	NA
	pERK1/2^{+}, ChAT^{+}, DBH^{+}	6	4	10	0.54	0.25	small

* 0.1 to < 0.3 – small, 0.3 to < 0.5 – moderate, and > or = 0.5 – large
